# Mesenchymal stem cell‐derived exosomes mitigate amyloid β‐induced retinal toxicity: Insights from rat model and cellular studies

**DOI:** 10.1002/jex2.70024

**Published:** 2025-01-22

**Authors:** Amanda Qarawani, Efrat Naaman, Rony Ben‐Zvi Elimelech, Michal Harel, Shahaf Sigal‐Dror, Tali Ben‐Zur, Tamar Ziv, Daniel Offen, Shiri Zayit‐Soudry

**Affiliations:** ^1^ Ruth and Bruce Rappaport Faculty of Medicine Technion Israel Institute of Technology Haifa Israel; ^2^ Clinical Research Institute Rambam Health Care Campus Haifa Israel; ^3^ Department of Ophthalmology Rambam Health Care Campus Haifa Israel; ^4^ Department of Human Genetics and Biochemistry, School of Medicine, Felsenstein Medical Research Center Tel Aviv University Tel Aviv Israel; ^5^ The Smoler Proteomics Center Technion Israel Institute of Technology Haifa Israel; ^6^ Department of Ophthalmology, Rabin Medical Center Tel Aviv University Tel Aviv Israel

**Keywords:** age‐related macular degeneration, amyloid β, mesenchymal exosomes, retina

## Abstract

Amyloid β (Aβ) has emerged as a pathophysiological driver in age‐related macular degeneration (AMD), emphasizing its significance in the aetiology of this prevalent sight‐threatening condition. The multifaceted nature of AMD pathophysiology, presumably involving diverse retinal cascades, corresponds with the complexity of Aβ‐induced retinopathy. Therefore, targeting a broad array of pathogenic processes holds promise for therapeutic intervention in AMD‐associated retinal pathology. This study investigates the potential of exosomes derived from adipose tissue mesenchymal stem cells (AT‐MSC‐Exosomes) in alleviating Aβ‐induced retinotoxicity. Through intravitreal injections in wild‐type rats and RPE‐like cell culture experiments, we examined the protective effects of AT‐MSC‐Exosomes against Aβ42 retinotoxicity. Our findings reveal that pre‐treatment with AT‐MSC‐Exosomes enabled nearly‐intact retinal function in vivo and maintained retinal cell viability in vitro, evidenced by longitudinal electroretinography (ERG) and XTT proliferation assays, respectively. Fluorescent labelling demonstrated increased migration of AT‐MSC‐Exosomes towards retinal cells under conditions of amyloid‐related toxicity. Proteomic analysis indicated a decrease in the retinal levels of heat‐shock proteins activated by pathogenic Aβ fibrils following AT‐MSC‐Exosome treatment. Similarly, immunostaining highlighted the modulation of α‐crystallin expression in retinal astrocytes by AT‐MSC‐Exosomes. These results suggest the potential therapeutic relevance of AT‐MSC‐Exosomes in Aβ‐related retinal pathology, offering a promising avenue for future AMD treatment strategies.

## 1 INTRODUCTION

The pathogenesis of age‐related macular degeneration (AMD) is complex, involving several processes that ultimately lead to the degeneration of retinal cells. Among the pathways shown to be implicated are dysregulated inflammatory activation, increased oxidative stress, and impaired lipid and cholesterol metabolism (Lin et al., 2016). To date, despite significant research efforts, the predominant molecular events that play a direct causative role in the degenerative cascade remain undefined. Moreover, it is presumed that a complex interplay among various mechanisms ensuing in the retina underlies the initiation and progression of this multifactorial disease.

Accumulating evidence indicates that Amyloid‐β (Aβ) plays a significant role in the pathophysiology of AMD. Aβ constitutes a family of aggregation‐prone and neurotoxic peptides that are linked with brain pathologies such as Alzheimer's disease. Human studies identified Aβ as a significant constituent of drusen deposits forming under the macula in eyes with AMD (Anderson et al., 2004; Dentchev et al., 2003; Ong et al., 2019). Both in vitro and in vivo studies have conclusively demonstrated the retinotoxic nature of Aβ aggregates (Anderson et al., 2008; Bruban et al., 2009; Gupta et al., 2016; Naaman et al., 2020; Walsh et al., 2005; Yoshida et al., 2005). It was shown that subretinal injection of Aβ in mice prompted retinal cell degeneration and retinal pigment epithelial (RPE) atrophy, effectively replicating an AMD‐like phenotype (Liu et al., 2015; Prasad et al., 2017; Yoshida et al., 2005). A collection of experimental data indicates that several pathways known as essential phenomena in AMD including oxidative stress, dysregulation of autophagy, neuroinflammation (Hu et al., 2021; Kauppinen et al., 2016), and mitochondrial dysfunction (Gupta et al., 2016) were activated in the retina by excess Aβ, which is hence postulated as a potential trigger factor in the onset of the disease (Gupta et al., 2016). Thus, devising strategies to inhibit the toxic impact of Aβ in the retina has gained interest as an approach for Aβ‐mediated neurodegeneration in AMD. Considering the multifaceted nature of the detrimental effects in the retina and the likelihood of a complex interplay among various signalling pathways, it is proposed that simultaneously targeting a broad array of pathogenic mechanisms can provide effective protection from Aβ‐induced retinal injury. Accordingly, an ideal intervention for Aβ‐induced retinal degeneration should dampen the inflammatory response while concurrently supplying trophic support to injured neurons and retinal cells. This two‐pronged approach has the potential to significantly improve outcomes in patients with retinal disease associated with Aβ toxicity.

In recent years, exosomes derived from mesenchymal stem cells sourced from adipose tissue (known as AT‐MSC‐Exosomes) have garnered significant attention due to their robust therapeutic potential, including anti‐inflammatory, anti‐apoptotic, tissue‐repairing, neuroprotective, and immunomodulatory capabilities, which closely resemble the attributes of AT‐MSCs themselves (Kalluri & LeBleu, 2020). These tiny extracellular vesicles are rich in bioactive molecules, including microRNAs, proteins such as cytokines and immunoregulatory factors secreted by their parent cells. What makes AT‐MSC‐Exosomes particularly intriguing is their ability to influence multiple signalling pathways (Kalluri & LeBleu, 2020), demonstrating a profound capacity to alleviate inflammation and foster the survival of injured neurons and additional retinal cells (Mead & Tomarev, 2020). Based on these attributes, AT‐MSC‐exosomes were envisioned as future therapy to treat amyloid‐associated neurodegenerations.

Here, we provide compelling evidence that AT‐MSC‐exosomes effectively migrate to the injured area and alleviate retinal damage caused in vivo by acute exposure to Aβ42 aggregates in rats. This mitigation is likely achieved through their affinity to inflammatory regions and capacity to incorporate into injured retina cells, facilitating the delivery of beneficial biological mediators which probably actively preserved the retinal function. Additionally, the exosome treatment led to a reduction in the expression of molecular participants in amyloid‐mediated retinal heat shock response. Such observations demonstrate the immediate therapeutic promise of AT‐MSC‐Exosomes in the retina but also shed light on the mechanisms responsible for Aβ42‐mediated retinotoxicity. These insights pave the way for further investigation of AT‐MSC‐Exosomes as a promising therapeutic strategy for amyloid‐related retinal disease.

## 2 METHODS

### 2.1 Preparation of human MSC‐derived exosomes

AT‐MSC‐Exosomes were obtained from human adipose tissue‐derived mesenchymal stem cells (hAT‐MSCs) provided by Everzom (Paris, France). Their proprietary production method utilizes turbulence in a 10‐L GMP‐compliant bioreactor. Following a 2D culture phase in flasks, hAT‐MSC cells were transferred onto specialized microcarriers designed to induce turbulence‐based stimulation. Exosome production commenced 4 days later in a 5‐L serum‐free medium, employing turbulence stimulation over a 4‐h duration. Throughout the production process, cell viability was monitored and remained consistently above 95% until production ceased. Upon completion of the production cycle, the microcarriers were allowed to settle, facilitating the collection of both the culture medium and exosomes. The harvested exosomes underwent clarification via a 0.45 µm filter and were subsequently isolated and concentrated through tangential flow filtration (TFF) and ultracentrifugation (UC). The final pellet was resuspended in PBS to achieve a concentration of 5.90 × 10^11 particles per milliliter and aliquoted into 200 µL vials. Exosome concentrations at each stage were determined using the NTA Zetaview system from Particle Metrix.

### 2.2 Fluorescent labelling of h‐AT‐MSC‐derived exosomes

PKH‐67 (6 µL) (Green Fluorescent Cell Linker Kits, Sigma–Aldrich; Merck KGaA) was incubated with 100 µL of exosomes (equivalent to a total of 5 × 10^10 exosomes), along with 1 mL of Diluent C. The mixture was then incubated at room temperature for 5 min. To arrest the staining process, 2 mL of a 10% Bovine Serum Albumin solution was added. Subsequently, the PKH67‐labelled exosome mixture underwent centrifugation at 110,000 × *g* for 2 h at 4°C, in a total volume of 70 mL of phosphate‐buffered saline (PBS). The resulting pellet was resuspended in sterile PBS and subjected to filtration using an Amicon centrifugal filter with a 30 kDa Ultracel‐30 regenerated cellulose membrane, within a 0.5 mL Amicon filter (Millipore Corp., Bedford, MA). This step effectively eliminated any soluble protein contaminants. The purified exosomes were resuspended in 100 µL of sterile PBS.

### 2.3 Preparation of Aβ42 fibrils and biophysical characterization of the assemblies

Aβ42 preparations were formulated as previously described (Naaman et al., 2020). Briefly, Aβ42 (Bachem, Heidelberg, Germany) (500 µg) was dissolved in high‐grade 1,1,1,3,3,3‐hexafluoroisopropanol (HFIP) (Sigma–Aldrich) by sonication for 20 s followed by constant shaking at 15 rpm at 37°C for 90 min, after which the solution was divided into aliquots. Monomeric Aβ was precipitated via speedvac. To derive fibrillar assemblies, monomeric Aβ was dissolved in DMSO by sonication before PBS was added. This mixture was aged at 37°C without shaking for 48 h.

The formation of Aβ fibrils was verified by transmission electron microscopy (TEM). The samples were placed on a copper grid (400 mesh) covered by carbon‐stabilized Formvar film (EMS, BNFFA1000‐Cu). After 2 min, excess fluid was removed and the grids were negatively stained with a uranyless solution (EMS, 22409). Finally, excess fluid was removed, and the samples were viewed in a JEM 1400plus electron microscope operating at 80 kV.

### 2.4 Cell lines

ARPE‐19 cells were cultured in (Dulbecco's Modified Eagle Medium) DMEM F12 (Ham's) (1:1) medium supplemented with 10% fetal bovine serum (FBS) (Biological Industries, Israel) and 1% Penicillin‐Streptomycin (thermofisher). The cells were incubated at 37°C 5% CO_2_.

### 2.5 Viability assays

ARPE‐19 cells (5000 cells/well) were grown to confluence in 96‐well tissue microplates in DMEM/Nutrient Mixture F12 (Ham's) (1:1) medium (200 µL per well) and allowed to adhere overnight at 37°C. AT‐MSC‐derived exosomes at a final concentration of either 2.8E8 particles/100 µL or 1.4E8 particles/100 µL were added to each plate 1 h before the addition of Aβ fibrils formed in vitro as outlined above (Aβ solutions diluted 200‐fold). The viability of experimental wells was compared with wells treated with Aβ fibrils only. Medium with no exosomes and no amyloid assemblies was used as a negative control. At experimental wells, exosomes were added for 1 h prior to incubation with Aβ fibrils overnight at 37°C, whereas wells treated with Aβ fibrils only served as controls. Cell viability was assessed using the XTT Cell Proliferation Kit (Biological Industries, Beit‐Haemek, Israel) according to the manufacturer's protocol. Briefly, 50 µL of XTT solution was added to each of the 96 wells, followed by a 2‐h incubation at 37°C. Colour intensity was measured using an ELISA plate reader at 500 nm and background subtraction at 650 nm. The results represent four biological repeats; the data are presented as mean ± Standard deviation. Statistical analysis was performed using student's *t*‐test.

### 2.6 Cellular uptake studies

To determine whether the exosomes exhibit a preferential homing pattern in the presence of toxic amyloid assemblies, exosomes derived from AT‐MSC were labelled with the fluorescent dye PKH67. Cultured ARPE‐19 cells were grown to confluence on coverslips (Marienfeld, Germany) in growth medium in 24‐well plates. At experimental wells PKH‐labelled exosomes were added for 30 min prior to adding fibrillar Aβ preparations for an overnight incubation at 37°C, while other wells were treated with fibrillar Aβ only or with PKH‐labelled exosome solutions alone. The cells were then rinsed with PBS and fixed in 4% PFA for 10 min at room temperature, before being washed with cold PBS and treated with 0.25% Triton X‐100 for 10 min to allow cellular permeabilization. Blocking was performed using 1% Bovine serum albumin (BSA) (MP biomedicals) for 1 h at room temperature. The cells were stained using the Aβ 1–16 (6E10, Biolegend) monoclonal antibody diluted 1:200 in blocking solution overnight at 4°C. The cells were washed three times with PBS and donkey anti‐mouse IgG secondary antibody (Invitrogen) diluted 1:500 in blocking solution was added for 1 h at room temperature in the dark. Cells were washed three times and stained with 5 µg/mL DAPI for 10 min. The cover slips were mounted using 15 µL immuno‐mount aqueous medium (Thermo Scientific). Imaging was performed using LSM 700 inverted Zeiss confocal microscope (Oberkochen, Germany). Excitation/emission wavelengths were 365/445 nm for DAPI and 560/630 nm for secondary antibody. Analysis was performed using ImageJ. The quantified signal was defined as the fluorescent area per cell number in each image, results are represented in a bar plot normalized to the PKH‐labelled exosome only treatment group. A threshold was set, and the quantified signal was defined as the fluorescent area in each image. Four images of each treatment group were compared using a student's *t*‐test. Results are represented in a bar plot normalized to control.

### 2.7 Retinal studies in rats

Experimental Sprague–Dawley (SD) rats were housed under 12/12‐h light/dark cycles, with unrestricted access to food and water. All animals were treated in accordance with the ARVO statement for the Use of Animals in Ophthalmic and Vision Research and according to institutional guidelines. The Ethics Committee of the Ruth Rappaport Faculty of Medicine, Technion, approved the study protocol. Before each intravitreal injection or electrophysiological recording, the rats were anesthetized with intraperitoneal injection (0.5 mL/kg) of a mixture consisting of Ketamine hydrochloride (10 mg/mL), Acepromazine maleate 10% and Xylazine 2%. Cyclopentolate hydrochloride 1% was used for pupil dilation and Benoxinate HCl 0.4% was instilled for corneal anaesthesia.

At baseline, the experimental rats underwent intravitreal injection of AT‐MSC‐exosomes to the right eye at the same time as a vehicle solution was injected to the left eye. The procedure was repeated after 2 days, when a 2nd injection of AT‐MSC‐exosomes or vehicle was administered to the corresponding eye. After additional 3 days (5 days after baseline), fibrillar Aβ42 was injected to both eyes. For each intravitreal injection (10 µL), a 32‐gauge needle was gently inserted to the vitreous chamber 1 mm posterior to the limbus, guided by simultaneous indirect ophthalmoscopy. In each rat, ERG was performed at baseline as previously described (Naaman et al., 2020). The electrophysiological assessment was repeated 5 days after the injection of MSC‐exosomes and through 28 days following the injection of Aβ. After the last ERG recording session, the rats were sacrificed, and the retinas were prepared for ex vivo examination.

The ERG responses were simultaneously recorded from the experimental and control eyes of each rat under dark‐adapted conditions. The system setup included custom‐made software, differential amplifiers (Grass Instrument Company, USA) and a Ganzfeld light source (LKC Technologies, USA) which emitted full‐field light stimuli with a maximum strength of 5.76 cd‐s/m^2^. Corneal electrodes were used in conjunction with subcutaneous stainless‐steel reference and ground electrodes (Roland Consult, Germany). Identical flashes were administered at 10‐s intervals to elicit repeated signals.

### 2.8 Analysis of the electroretinographic response

For analysis of the ERG, for each response, the amplitudes of the a‐wave and the b‐wave from the experimental and control eyes were measured and the maximal amplitude, representing the maximal retinal function, was determined by mathematical fit to a hyperbolic function as previously described (Naaman et al., 2020). For each rat at each recording session, the ratios between the maximal amplitudes of the a‐wave and the b‐wave obtained from each eye (right vs. left) were calculated and used as a measure of their relative functional status.

To evaluate the integrity of the retinal ERG response originating from each eye and to delve into the underlying nature of the reflected retinal function, we examined the correlation between the a‐wave and the subsequent b‐wave amplitudes in each response. The a‐wave is indicative of the photoreceptor function, while the b‐wave is generated by post‐receptoral cells, predominantly bipolar cells. Typically, there exists a certain interdependence between the amplitude of the b‐wave and the preceding a‐wave, as the photoreceptor response triggers the subsequent reaction of retinal cells (Asi & Perlman, 1992). When the transmission between photoreceptors and post‐receptoral cells is intact, the relationship between the a‐wave and the b‐wave maintains a consistent pattern. Deviations from this anticipated range can serve as indicators of dysfunction within specific retinal components, distinguishing between impairments that primarily affect the photoreceptors versus those affecting post‐receptoral components (Asi & Perlman, 1992). The collection of b‐a wave ratio data points obtained from each eye at the baseline, which represents the typical retinal physiology, served as the control dataset against which the ratios acquired at subsequent timepoints following the interventions were compared.

### 2.9 Proteomic analysis

For proteomic analysis, rats (*n* = 3) were treated as described above. In each rat, the experimental eye was injected with AT‐MSC‐derived exosome, and the control eye received vehicle alone 2 days before fibrillar Aβ was administered to both eyes. The rats were sacrificed 6 days after Aβ injection. Eyes were enucleated and extraocular tissue was carefully dissected. The anterior segment was discarded around the ora serrata. The eyes were transferred to DMEM, when the neurosensory retinas were delicately extracted and frozen immediately in liquid nitrogen.

The proteins were extracted from the retina in 9 M Urea, 400 mM Ammonium bicarbonate and 10 mM DTT following grinding (Omni TH Tissue Homogenizer) and two cycles of sonication. A total of 20 µg protein from each sample were reduced with 3 mM DTT (60°C for 30 min), modified with 9 mM iodoacetamide in 400 mM ammonium bicarbonate (in the dark, room temperature for 30 min) and digested in 1 M Urea, 50 mM ammonium bicarbonate with modified trypsin (Promega) at a 1:50 enzyme‐to‐substrate ratio, overnight at 37°C. An additional second trypsinization was done for 4 h.

### 2.10 Mass spectrometry analysis

The tryptic peptides were desalted using C18 stagetips dried and re‐suspended in 0.1% Formic acid. The peptides were resolved by reverse‐phase chromatography on 0.075 × 180‐mm fused silica capillaries (J&W) packed with Reprosil reversed phase material (Dr Maisch GmbH, Germany). The peptides were eluted with a different concentration of Acetonitrile with 0.1% of formic acid: a linear 180‐min gradient of 5%–28% acetonitrile followed by a 15 min gradient of 28%–95% and 25 min at 95% acetonitrile with 0.1% formic acid in water at flow rates of 0.15 µL/min.

Mass spectrometry of the retinal samples was performed by Q Executive HFX mass spectrometer (Thermo) in a positive mode (m/z 350–1200, resolution 120,000 for MS1 and 15,000 for MS2) using repetitively full MS scan followed by collision‐induced dissociation (HCD, at 27 normalized collision energy) of the 30 most dominant ions (charges) selected from the first MS scan. The AGC settings were 3 × 106 for the full MS and 1 × 105 for the MS/MS scans. A dynamic exclusion list was enabled with an exclusion duration of 20 seconds. The mass spectrometry data was analyzed using the MaxQuant software 2.1.3.0 (Cox et al., 2014) for peak picking and identification using the Andromeda search engine, searching against the Rattus norvegicus proteome from the Uniprot database with a mass tolerance of 6 ppm for the precursor masses and the fragment ions. Oxidation on methionine and protein N‐terminus acetylation were accepted as variable modifications and carbamidomethyl on cysteine was accepted as static modifications. A Minimal peptide length was set to seven amino acids and a maximum of two miscleavages was allowed. The data was quantified by label‐free analysis using the same software. Peptide‐ and protein‐level false discovery rates (FDRs) were filtered to 1% using the target‐decoy strategy. Protein tables were filtered to eliminate the identifications from the reverse database, and common contaminants and single peptide identifications (Tyanova et al., 2016).

The data was quantified by label‐free analysis using the same software. Statistical analysis of the identification and quantization results was done using Perseus 1.6.2.2 software (Mathias Mann's group).

To identify changes in the protein intensities following the treatment, we performed a T‐test between the groups. Since the experimental treatment did not uniformly affect each animal, only two proteins showed a reduction and two others were elevated, all with a *p*‐value below 0.05‐ and a 2‐fold change difference for the entire group of rats (*n* = 3). To gain a deeper understanding of more nuanced changes, we also examined all the proteins that exhibited at least a 2‐fold change difference in at least two repeats, even if no change was seen in the third. Annotation enrichment analysis was generated using the STRING database version 12.0, and annotation terms were filtered for high strength (>0.700).

### 2.11 Retinal uptake of exosomes

To investigate the migration of AT‐MSC‐exosomes to retinal injury sites, rats were injected with Aβ fibrils in their right (experimental) eye and vehicle in the left (control) eye. ERG was performed at baseline and 7 days post injection to assure the functional retinal status. Upon confirmation of the development of Aβ‐mediated retinal dysfunction, both eyes were injected with fluorescent‐labelled AT‐MSC‐exosomes tagged with PKH67 (10 µL). The rats were kept in dim‐light and were sacrificed 1 h post exosome injection when the retinal tissue was prepared for further studies.

### 2.12 Immunofluorescence of rat retinal sections

Eyes enucleated 9 days post fibrillar Aβ injection and an hour post PKH‐labelled exosome injection were fixated in PFA 4% for an hour followed by a treatment with a gradient of sucrose solutions. The eyecups were then frozen in OCT (Thermo Fisher Scientific) before being sectioned by cryostat to 16 µm‐thick slices. For the staining, the tissue sections were permeabilized in 1% Triton X‐100 (Sigma–Aldrich) and then treated with 10% FBS blocking solution (Biological industries, Israel). The sections were subsequently incubated overnight at 4°C with a primary anti‐GFAP antibody (GA5, Invitrogen) diluted 1:1000 or alternatively with Amyloid β antibody (6E10, Biolegend) diluted 1:500. Additionally, retinal sections from eyes enucleated 9 days after the second exosome injection and 7 days post fibrillar Aβ injection were immune‐stained targeting the B‐subunit of α‐crystallin antibody (ab76467, Abcam) diluted 1:250. For secondary antibodies, Alexa Fluor 594‐labelled donkey anti‐mouse IgG was used at 1:500 dilution (A21203, Invitrogen) and Alexa Fluor 488‐labelled goat anti‐rabbit IgG (ab150077, Abcam) was used at 1:500 dilution. Nuclei were stained with DAPI. As a control, sections from experimental eyes were stained with secondary antibody only. Samples were examined using a Zeiss (Oberkochen, Germany) confocal LSM 700 microscope. Signal quantification was performed using ImageJ. A threshold was set, and the quantified signal was defined as the fluorescent area in each image. Six images of each treatment group were compared using student's *t*‐test. Results are represented in a bar plot normalized to control.

### 2.13 Statistical analysis

All statistical calculations were performed using Microsoft Excel version 7 (Microsoft Corporation, NY, USA). Comparison between the different study groups was done using Student *t*‐test. All results were reported as a mean ± standard deviation. A probability value (*p* value) less than 0.05 was considered statistically significant.

## 3 RESULTS

### 3.1 AT‐MSC‐derived exosomes protect against Aβ42‐induced cytotoxicity in ARPE‐19 cells

**FIGURE 1:**
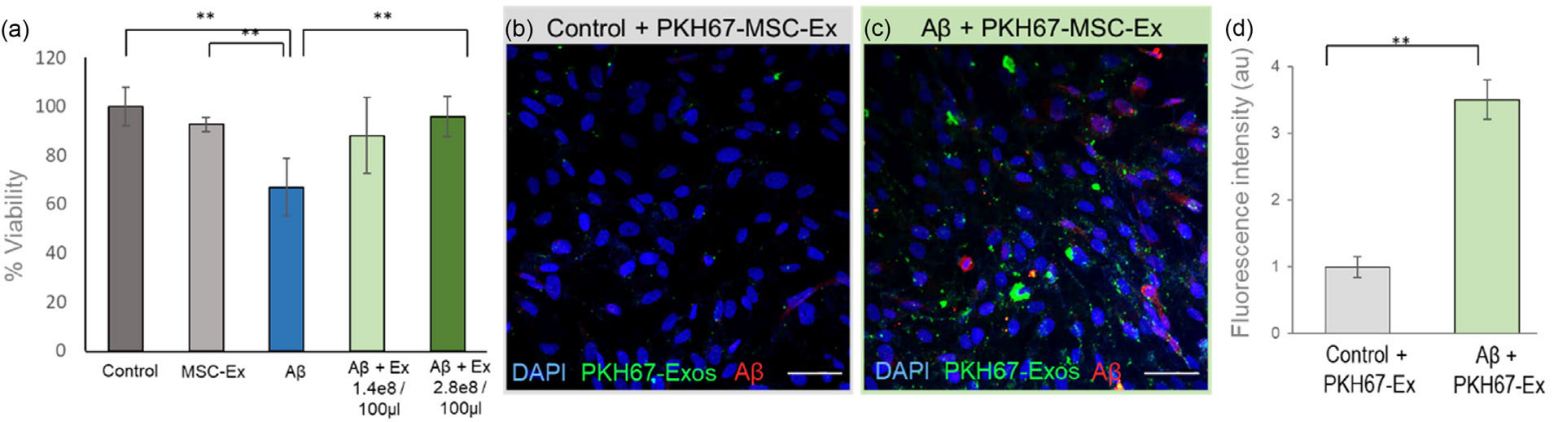
MSC‐exosomes show increased migration and ameliorate the toxicity of Aβ in vitro. (a) ARPE‐19 cells were treated with vehicle alone (dark gray column), Aβ fibrils only (blue column), MSC exosomes only (light gray column) and MSC exosomes at two concentrations (1.4E8 particles/100 µL, light green, 2.8E8 particles/100 µL, dark green) prior to Aβ fibril administration. The XTT proliferation assay was used to determine the cellular viability following each treatment. Only the higher exosome concentration (2.8E8 particles/100 µL) was neuroprotective, yielding cell counts equivalent to controls. ** Indicate *p* value < 0.05. (b and c) ARPE‐19 cells were cultured with PKH67‐labelled exosomes 30 min before incubation with vehicle solutions (b) or fibrillar Aβ preparations (c). Cells were stained for the presence of Aβ using a specific antibody. Calibration bars: 50 µm. (d) Signal intensity measurement. The data are presented as mean normalized to control ± normalized s.e.m. (*n* = 4, **p < 0.05).

Aβ42 solutions were incubated, and TEM microscopy was employed to characterize the morphology of the derived supramolecular assemblies and confirm their fibrillar structure (Figure S1). To examine the potential protective role of AT‐MSC‐derived exosomes against Aβ cytotoxicity in cultured cells, the XTT cell viability assay was used. ARPE‐19 cells were cultured with cell growth media containing Aβ42 fibrils with or without prior administration of AT‐MSC‐derived exosomes (final concentration of either 2.8E8 particles/100 µL or 1.4E8 particles/100 µL in treated wells) for 1 h. In comparison to vehicle‐only treated controls, exposure to AT‐MSC‐exosomes alone had no discernible effect on the cell counts, but fibrillar Aβ significantly reduced the cell viability by 14% (Figure 1a). In contrast, the toxic effect of fibrillar Aβ assemblies was substantially reduced in wells that were preincubated with AT‐MSC‐derived exosomes at 2.8E8 particles/100 µL, wherein cell viability remained unaffected at 94% (Figure 1a). Hence, AT‐MSC‐derived exosomes inhibit the cytotoxicity of fibrillar Aβ42 in vitro.

### 3.2 The internalization of AT‐MSC‐derived exosomes is increased in the presence of fibrillar Aβ42

To investigate the intracellular presence of exosomes under various conditions of amyloid exposure, we employed fluorescence‐labelled exosomes along with Aβ‐specific antibody in ARPE‐19 cells. In cell cultures treated exclusively with PKH‐labelled AT‐MSC‐derived exosomes, only a faint positive signal was observed (Figure 1b), indicating minimal exosome incorporation under standard conditions. In contrast, no discernible fluorescent signal was detected in cells treated with the vehicle alone, without the addition of exosomes (data not shown). When cells were pre‐exposed to AT‐MSC‐derived PKH‐tagged exosomes and then treated overnight with fibrillar Aβ42, a striking 350% increase in positive fluorescent signal was observed (*p* = 0.012), indicating a substantial increase in exosomes migration towards affected cells (Figure 1c,d). The exosomal markers were predominantly noted in regions displaying Aβ‐positive staining, clearly signifying an increased migration of exosomes towards locations linked to the amyloid‐induced pathology.

### 3.3 AT‐MSC‐derived exosomes protect retinal function against Aβ42 toxic effect in vivo

**FIGURE 2:**
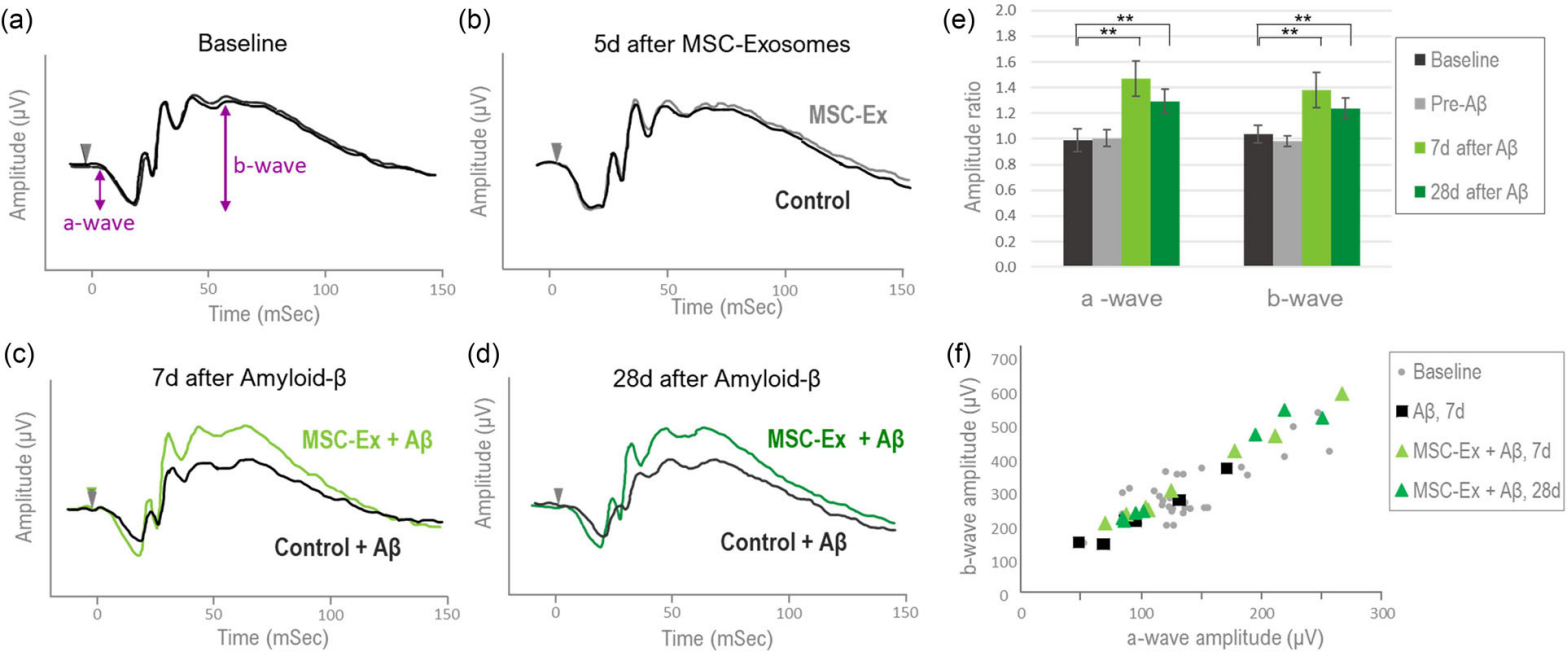
AT‐MSC‐derived exosomes protect the retinal function from Aβ toxicity. (a–d) Representative ERG responses from a single rat treated with intravitreal exosome injection in the right eye prior to fibrillar Aβ42 administration to both eyes. Each frame shows overlays of the tracings obtained simultaneously from the right (colored lines) and left eyes (black traces). The ERG a‐wave and the b‐wave are denoted in frame a. (e) Mean ratios between the ERG amplitudes from the MSc‐exosomes treated eyes versus the amplitudes from their fellow eyes treated with Aβ without prior injection of exosomes (*N* = 5 rats). While at baseline (a) as well as following injection of exosomes to one eye only (b) the ERG responses from both eyes are highly similar, at 7 days (c) and 28 days after Aβ injection (d) the responses from the right eyes are higher compared with those from the left eye, deriving increased right/left amplitude ratios. ** Indicate *p* value < 0.05. (f) The ratio between the amplitude of the b‐wave versus that of the a‐wave was assessed for each response. The grey markers indicate the measures obtained at baseline which reflect the normal ERG configuration. The black markers represent the responses obtained after injection of Aβ, and the green markers represent the measures from the experimental eyes treated with exosomes prior to injection of amyloid fibrils.

To determine the protective effect of AT‐MSC‐derived exosomes against fibrillar Aβ42‐mediated retinotoxicity in vivo, the retinal function was examined in rats. AT‐MSC‐derived exosomes were administered twice to the right eye of wild‐type rats (*n* = 5), while at the same time the left eye of each rat was injected with a control solution, followed by an intravitreal injection of retinotoxic Aβ fibrils into both eyes 2 days later. ERG testing was conducted at baseline, 5 days after the first injection of exosomes (and prior to injection of Aβ), as well as 7, 14 and 28‐days after Aβ42 administration. Notably, the administration of AT‐MSC‐derived exosomes did not affect the retinal function, as the ERG responses remained intact and unchanged after the second exosome injection compared to the baseline measurements (Figure 2a,b). However, following administration of fibrillar Aβ42 all rats exhibited impaired retinal function in the control eye treated with Aβ42 alone (Figure 2c, dark trace). The decline in retinal function was evident by decreased ERG amplitudes as early as 7 days after the injection, which remained attenuated through 28 days of follow‐up (Figure 2d, dark trace).

In contrast, eyes preconditioned with AT‐MSC‐derived exosomes prior to administration of Aβ42 demonstrated intact ERG responses with amplitudes nearly resembling normal levels (Figure 2c,d, green traces). The marked difference between the responses from the eyes preconditioned with AT‐MSC‐exosomes prior to Aβ exposure and their fellow eyes treated with Aβ alone persisted through 28 days (Figure 2d).

To comprehensively analyze the retinal function and derive insight into the magnitude of the protective effect provided by the MSC‐exosomes, we employed a quantitative approach. For each recording session of each rat, we determined the maximal amplitudes of the ERG a‐wave and b‐wave using a hyperbolic function (Hood & Birch, 1992). At each designated time point, the ratio between the maximal amplitudes obtained from the control eye and the experimental eye of each rat was calculated. Under normal physiological conditions when the retinal function is similar in both eyes, the amplitudes of the ERG are typically nearly identical, resulting in a ratio close to 1. Accordingly, at baseline as well as after injection of AT‐MSC‐exosomes, the mean amplitude ratios derived for the entire group of rats were approximately 1 for both the a‐wave and the b‐wave components (Figure 2e). In contrast, following the Aβ42 injection, there was a substantial divergence between the ERG responses in each rat, leading to an increased amplitude ratio favouring the eyes pre‐treated with exosomes before Aβ injection (Figure 2e, green bars). The mean values ranged between (1.29–1.47) for the a‐wave and (1.25–1.55) for the b‐wave, clearly indicating the protective effects of MSC‐derived exosome against Aβ42‐mediated retinal dysfunction. The differences in mean amplitude ratio were statistically significant when compared to baseline starting at Day 7 and extending through 28 days post Aβ42 injection (*p* < 0.05 for all), (Figure 2e).

To further elucidate the ERG configuration and the reflective state of retinal function, we investigated the relationship between the amplitudes of the b‐wave and the preceding a‐wave for each response. The ratios derived from baseline measurements of all eyes served as the reference normal controls, depicted as grey markers in Figure 2f. Seven days following the injection of Aβ, eyes treated solely with Aβ exhibited ratios that closely approached the lower boundary of the normal range (Figure 2f, black markers), suggesting some impairment in the photoreceptors response and potential attenuation of the signal transmission to post‐receptoral cells. In contrast, eyes that received MSC‐exosomes before Aβ injection demonstrated ratios that, while remaining within the normal range, were positioned near the upper boundary (Figure 2f, green markers). These observed patterns suggest a combined rescue effect on both retinal photoreceptors and post‐receptoral retinal neurons imparted by the MSC‐exosomes.

### 3.4 Enhanced uptake of AT‐MSC‐derived exosomes in Aβ42‐ treated retinas

**FIGURE 3:**
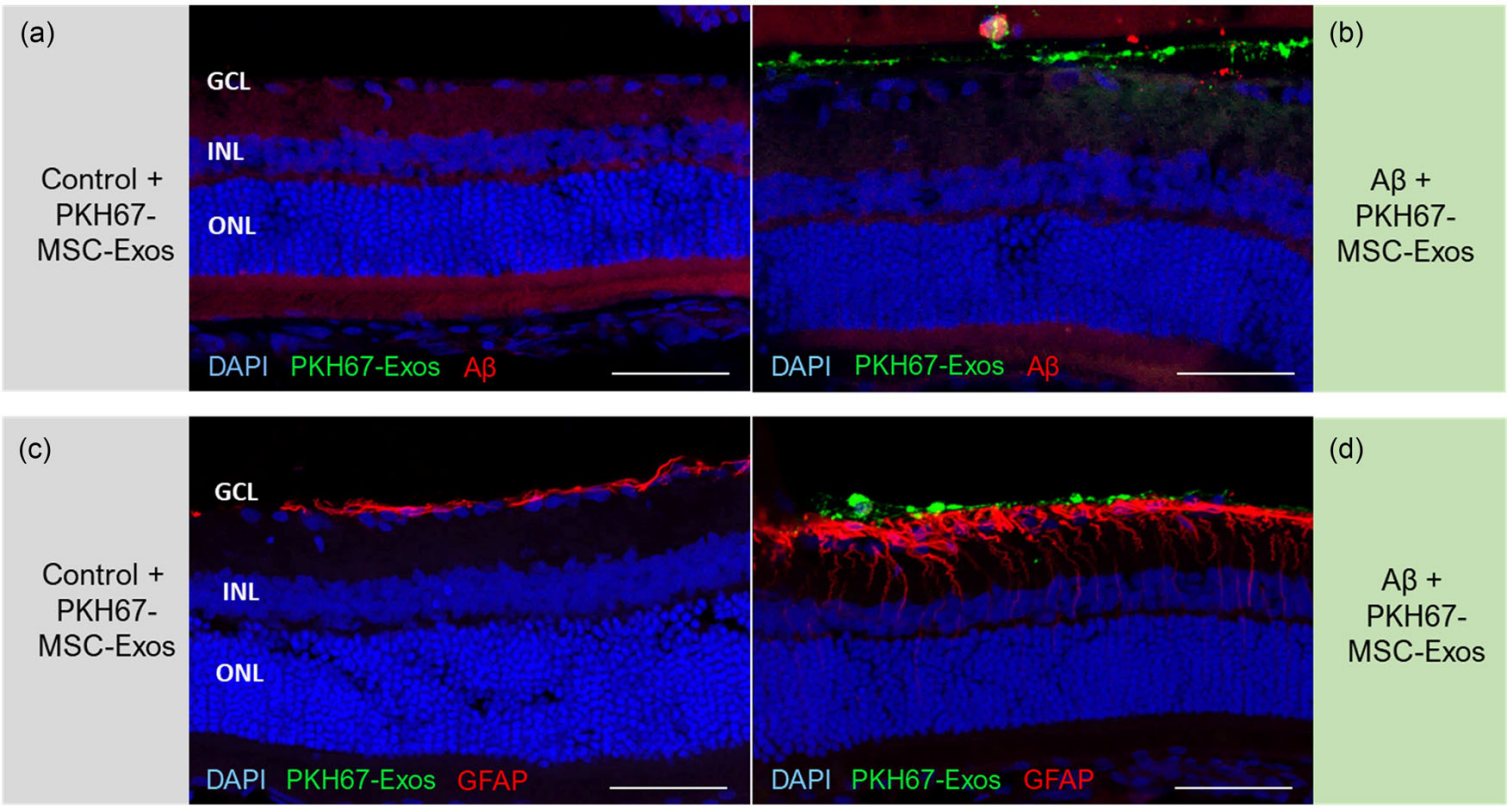
Localization of exosomes derived from AT‐MSCs toward the retina following exposure to fibrillar Amyloid‐β (Aβ). Incorporation of the injected Aβ assemblies into the retina is confirmed by Aβ staining 9 days post amyloid injection (a and b), while Glial Fibrillary Acidic Protein (GFAP) staining serves as an indicator of glial cell activation (c and d). Retinal sections from (a) a control eye treated with the vehicle and (b) an experimental eye treated with Aβ42, respectively, prior to the administration of PKH67‐labeled MSC‐derived exosomes. Specific Aβ staining was expectedly negative in the control eye (a) but confirmed the presence of Aβ in the inner retina in the experimental eye (b). In eyes exposed to Aβ (b and d), PKH‐labelled AT‐MSC‐derived exosomes are observed within the ganglion cell layer and inner nuclear layer, and GFAP staining reveals Müller cell activation (d). Conversely, in control eyes (a and c), the green PKH67 signal reflecting AT‐MSC‐derived exosomes is absent from the retina, and positive GFAP staining indicates astrocytic patterns but not the activation of Müller cells. The results represent three biological repeats for each study. Calibration bar: 50 µm.

To investigate the distribution of exosomes in intact and damaged retinas in an in vivo context, we employed fluorescent‐labelled AT‐MSC‐derived exosomes (Figure 3). Rats (*n* = 5) were treated with fibrillar Aβ42 in the right eye and vehicle in the left eye and ERG was subsequently performed 7 days later to assess for the occurrence of amyloid‐mediated retinal dysfunction in the right eye. Following verification of the retinotoxic impact of Aβ42, each rat received an intravitreal injection of PKH‐labelled AT‐MSC‐derived exosomes to both eyes and was sacrificed 1 h later. To ascertain the retinal localization of the injected Aβ fibrils and to examine the relationship between the amyloid uptake and the presence of fluorescent‐exosomes markers, staining with Aβ specific antibody was employed (Figure 3). In retinal sections from the control eyes treated with vehicle followed by PKH67‐labelled MSC‐derived exosomes, only negligible exosome‐related (green) fluorescence was observed along the inner retinal layers (Figure 3a). Expectedly, Aβ staining was negative in these eyes. In contrast, retinal sections from the eyes treated with Aβ42 aggregates prior to injection of PKH67‐tagged AT‐MSC‐derived exosomes manifested markedly distinct patterns of fluorescence. First, a positive Aβ (red) signal was noted in all samples, confirming the retinal localization of the injected fibrils. Additionally, a robust PKH (green) signal was detected, spanning the ganglion cell layer and the inner plexiform layer across all specimens (Figure 3b), confirming that exosomes migrated to retinas affected by pathogenic amyloid assemblies. These findings indicate increased migration dynamics of MSC‐derived exosomes from the vitreous to the retina in response to the toxic effects of fibrillar Aβ42 assemblies induced in an in vivo setting.

### 3.5 AT‐MSC‐derived exosome treatment alters the expression of GFAP glial activation marker

To obtain additional insight into the protective effect against amyloid‐related retinopathy provided by MSC‐exosomes, immunofluorescence analysis using the glial fibrillary acidic protein (GFAP) specific antibody was performed on the same retinal sections. GFAP is normally expressed by retinal astrocytes, but in response to retinal damage, this protein becomes upregulated in retinal Müller glia cells (Dyer & Cepko, 2000). Consistent with the remarkable differences between the functional status of retinas treated with retinotoxic Aβ assemblies with or without prior administration of MSC‐derived exosomes, distinct GFAP staining patterns were seen. In control eyes following injection of MSC‐exosomes coupled with injection of vehicle, a weak positive signal was seen merely in retinal astrocytes, indicating physiological staining and confirming the absence of significant glial activation (Figure 3c). Conversely, in eyes treated with fibrillar Aβ42 prior to injection of the PKH‐tagged exosomes, intense GFAP immunoreactivity was seen in all samples from all eyes (Figure 3d). The positive signal was noted along vertical structures corresponding to Müller cells, confirming retinal stress and glial activation process associated with Aβ exposure.

### 3.6 Proteomic analysis of neurosensory retina

To better understand the molecular details of the exosome‐mediated preservation of retinal function noted in vivo, proteomic profiling using mass spectrometry was performed. Proteomic profiles were compared between experimental eyes conditioned with MSC‐derived exosomes prior to fibrillar Aβ injection and control eyes treated with the retinotoxic Aβ assemblies alone.

In total, 3940 proteins were successfully identified and quantified using the MaxQuant platform. Among these proteins, 32 were defined as upregulated and 21 were downregulated in eyes treated with unopposed Aβ compared to their fellow eyes treated with MSC‐exosomes prior to Aβ exposure. Following a rigorous protein interaction analysis, the most enriched term was ‘Lens development in camera‐type eye’ (GOBP0002088) with strength 1.84 and *p*‐value 1.20E‐14. Out of the 12 proteins that were annotated with this term, 11 dysregulated proteins were identified from the α‐, β‐ and γ‐ crystallin family (Table S1).

### 3.7 Modulation of small heat shock protein expression in the retina

**FIGURE 4:**
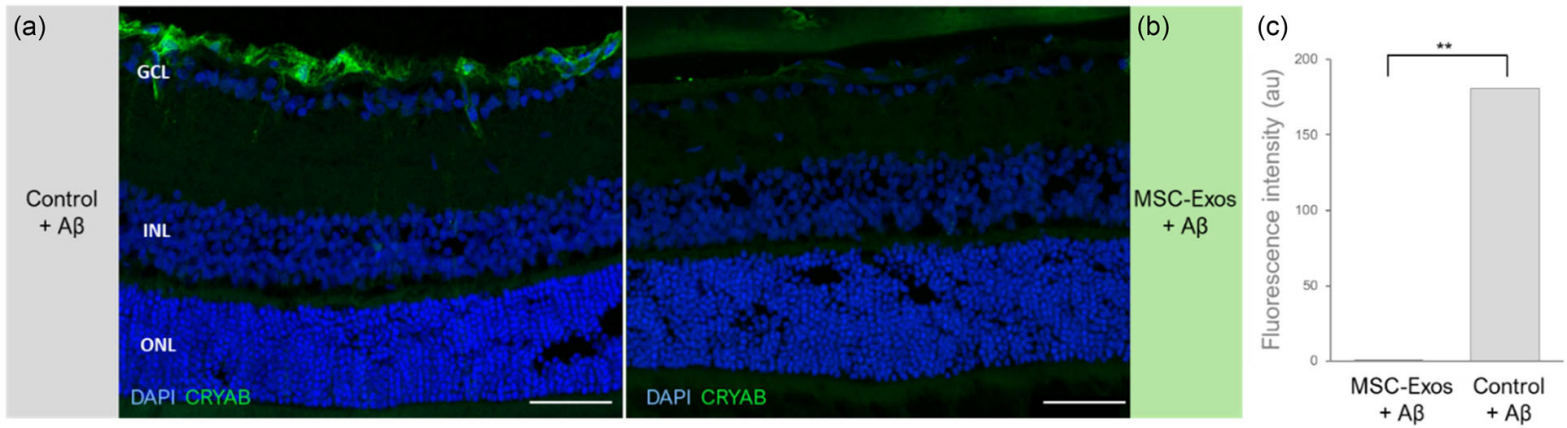
Immunodetection of the B‐subunit of α‐crystallin (CRYAB) in retinal astrocytes in response to Aβ42. (a) In rat eyes treated with fibrillar Aβ42, CRYAB expression is identified in branched cells along the inner retinal layers. Similar expression profiles were seen in all biological replicates (*n* = 3). (b) Illustrative retinal section from a rat eye treated with MSC‐exosomes prior to injection of the amyloid fibrils. No positive staining for CRYAB was detected. Calibration bars: 50 µm. Retinal layers indicated: Ganglion cell layer (GCL), Inner nuclear layer (INL), Outer nuclear layer (ONL). (c) Quantitative analysis confirms that immunoreactivity for CRYAB following Aβ42 injection was dramatically reduced in eyes pre‐conditioned with MSC‐Exosomes (*n* = 3 rats). ** Indicate *p* value < 0.05.

To validate the proteomic results and to confirm the differential expression of the small heat shock crystallin proteins in response to Aβ, with or without prior conditioning with exosomes, immunostaining targeting the α‐crystallin B‐subunit was employed. In eyes injected with fibrillar Aβ42, distinct staining was identified in branched cells along the inner retina, consistent with retinal astrocytes (Figure 4a). In contrast, following the pre‐administration of exosomes, no positive signal was detected in all treated eyes (Figure 4b). Quantitative analysis confirmed a significant reduction in the fluorescent signal intensity between these conditions. These results corroborate the inhibition of retinal glial cell activation provoked by retinotoxic Aβ in the presence of MSC‐derived exosomes.

## 4 DISCUSSION

To date, most conventional treatments for AMD have failed to provide a disease‐modifying approach and arrest the degeneration of the retina cells. The pathophysiological basis of AMD is intricate and multifactorial, involving a range of interrelated effectors and various mechanisms. Among the putative contributors to AMD progression, Aβ has been identified as a hypothetical mediator, with an intriguing capacity to contribute to diverse retinal malfunctions which are associated with AMD. For example, Aβ assemblies have been shown to co‐localize with complement factors and to promote a chronic retinal state of complement pathway activation (Anderson et al., 2004; de Almeida Torres et al., 2021; Liu et al., 2013). Aβ can further elicit microglial response, oxidative stress and proinflammatory activity (Chen et al., 2016; Lei et al., 2017; Liu et al., 2013), thereby accelerating photoreceptor and retinal cell stress and apoptosis (Sun et al., 2020; Tsao et al., 2018; Wang et al., 2017; Wu et al., 2021). In essence, Aβ may serve as an intermediary link potentially connecting the various cascades occurring in the retina in AMD. Thus, a rational strategy for enhancing therapeutic potential in the state of Aβ‐inflicted retinal pathogenicity may benefit from a combined approach that targets multiple mechanisms. Such an approach may form a potential basis for future developments in AMD therapies.

In the present study, we harnessed AT‐MSC‐derived exosomes to treat amyloid‐related retinal pathology, revealing their beneficial properties in vitro and in vivo. The exosomes effectively migrated into recipient retina cells and preserved their integrity in the presence of toxic Aβ fibrils. These protective effects correlated with the reduction of retinal glial activation and the downregulation of crystallin mediators of the heat‐shock response, which were triggered by fibrillar Aβ in the absence of exosomes. Our results align with previous research which demonstrated that MSC‐derived exosomes induced diverse beneficial effects in preclinical models of Alzheimer's disease and specifically protected against neuronal damage induced by Aβ, with improved cognitive behaviour, learning and memory capabilities in mice (Wang et al., 2018). The underlying mechanisms were attributed to enhanced neuronal viability, improved neuronal function and rescue of synaptic impairment (Bodart‐Santos et al., 2019; Cui et al., 2018; Lee et al., 2018; Reza‐Zaldivar et al., 2019; Wang & Yang, 2021; Wang et al., 2018).

We noted an increased presence of exosomes in RPE‐like and retina cells inflicted by Aβ. These observations are consistent with previous studies of neurodegeneration of the brain which reported that MSC‐exosomes specifically targeted and accumulated in pathologically‐relevant brain regions in preclinical models (Perets et al., 2019). The retention of exosomes was detected to last in sites of brain pathology and loci of neuroinflammation for up to 96 h after administration, in clear contrast to their behaviour in healthy controls, where they showed dispersed migration pattern and were largely cleared by 24 h (Perets et al., 2019). In agreement, our findings show that AT‐MSC‐derived exosomes exhibit retinal tropism, facilitating their migration from the vitreous in vivo particularly under conditions of functional retinal cell damage and glial activation. Their nanoscale dimensions and bilipid membrane composition provide MSC‐derived exosomes with a unique ability to efficiently penetrate biological impediments (Heidarzadeh et al., 2021). Our results confirm their capacity to successfully breach the ocular barriers presented by the vitreous and the inner limiting membrane (ILM) under conditions of amyloid‐pathogenicity and localize within the retina. This allowed the efficient delivery of their biologically active cargo which influenced the global retinal processes, enabling favourable retinal effects that persisted for 28 days.

The molecular details of the mechanism of action of MSC‐exosomes in amyloid‐related neuro‐pathologies are yet to be deciphered in depth. Several investigations reported that MSCs‐derived exosomes reduced the levels of Aβ and pro‐apoptotic protein content in vitro and in vivo (Bodart‐Santos et al., 2019; Lee et al., 2018; Reza‐Zaldivar et al., 2019; Wang & Yang, 2021). Our data indicate the localization of the injected fibrils in distinct regions of the retina which contrasted with the global impairment of retinal function reflected in the ERG responses from Aβ‐treated eyes. The diffuse retinal dysfunction exerted by Aβ may be potentially attributed to the widespread release of toxic entities from the fibrillar assemblies, possibly via mechanisms of secondary nucleation, versus the activation of pathological cascades diffusely affecting the retina. This observation aligns with our ex vivo findings demonstrating the broad dispersion of PKH‐labelled exosomes in the presence of retinal Aβ toxicity. Consequently, it is suggested that the primary effects of the exosomes are not necessarily based on direct targeting of the amyloid aggregates, but instead involve the modulation of secondary effects triggered by Aβ within the retina. This finding is consistent with prior studies that underscore the significance of MSC‐derived extracellular vesicles in ameliorating Alzheimer's disease models via the regulation of microglial activation and neuroinflammation (Ding et al., 2018; Losurdo et al., 2020). In vitro studies conducted in cell cultures revealed that MSC‐derived exosomes diminished reactive oxygen species (ROS) formation and maintained synaptic density following exposure to oligomeric Aβ (Gonçalves et al., 2023), with comparable outcomes observed in rat hippocampal neurons (Bodart‐Santos et al., 2019). Another study demonstrated that MSC‐derived exosomes inhibited Aβ‐induced macrophage activation in BV cells (Kaniowska et al., 2022). Our ERG results indicate that both photoreceptors and retinal neurons were effectively rescued by the exosomes, which supports the notion that exosomes may induce broad anti‐inflammatory or anti‐stress reactions, which can impact various susceptible retinal cells without necessarily being specific to particular cell types. Accordingly, the positive impact of exosomes has been shown to protect the retina against various mechanisms of damage involving extensive pathogenic processes. For example, intravitreal introduction of MSC‐derived exosomes 24 h after induction of retinal ischemia in rats led to substantial improvements in functional recovery, a reduction in neuro‐inflammation and a decrease in apoptosis (Mathew et al., 2019). Notably, the exosomes were effectively internalized by retinal neurons, retinal ganglion cells and microglia.

To elucidate the nature of the observed protective effects, we conducted proteomic analysis. We found elevated levels of several crystallin proteins in control retinas treated with fibrillar Aβ alone. Crystallins are key members of the small heat shock superfamily of proteins (sHSP) (Kannan et al., 2012; Sreekumar et al., 2010). In the retina, α‐Crystallins are secreted apically by the RPE in response to various physiological and pathological conditions (Kannan et al., 2012; Sreekumar et al., 2010), and were found to be abundantly expressed among drusen constituents in eyes with AMD (Crabb, 2014). Recently, several biological activities were attributed to the presence of α‐crystallin in the extracellular space. Specifically, the B‐subunit of α‐crystallin (αB‐crystallin) provides cellular protective functions by inhibiting apoptotic cell death, mitigating oxidative stress and providing anti‐inflammatory effects (Dou et al., 2012; Sreekumar et al., 2012; Yaung et al., 2007). In the context of amyloid toxicity, crystallins possess chaperone‐like functions and were found to exhibit neuroprotective properties against Aβ. The α‐ and β‐crystallin isoforms can interact with Aβ, inhibiting fibril elongation and reducing its propensity to release pathogenic species via secondary nucleation (Selig et al., 2022; Shammas et al., 2011; Sharma et al., 1997). Thus, the rise in these proteins in control retinas signifies the intrinsic defence response to the amyloid fibrils. Such protective mechanisms were not expressed in retinas treated with AT‐MSC‐exosomes prior to Aβ injection, thereby reflecting a potential reduction in the stress imposed by amyloid pathology, which was facilitated by the action of the exosomes. The specific localization of the αB‐crystallin in retinal astrocytes observed in eyes treated with Aβ fibrils, coupled with the morphology of the glia cells, indicates their activated state and possible migration in response to the amyloid insult. Indeed, our results are in line with previous reports showing αB‐crystallin upregulation in astrocytes in response to various pathogenic states, including pro‐inflammatory conditions as well as in models of Alzheimer's disease (Hochberg et al., 2014; Iwaki et al., 1992; Renkawek et al., 1999, 1994). The increase in crystallins in such states of reactive gliosis can occur either as a defence mechanism against apoptosis, aimed to provide immune support, or indicates their role in regulating the function of the reactive astrocytes (Alge et al., 2002; Nagaraj et al., 2005; Ousman et al., 2007). Although the details of mechanisms through which the exosomes prevented the expression of crystallins and the way it is linked with the mitigation of Aβ toxicity in the retina merit further investigation, these findings highlight the intricate interplay between the amyloid response and retinal neurodegeneration.

Collectively, our findings offer a new perspective on amyloid‐related pathologies related to AMD and present a potential avenue for treating this currently therapy‐lacking, debilitating condition. Notably, this study represents the pioneering investigation into the substantial impact of MSC‐exosomes in ameliorating retinal degeneration. In recent years, modulation of retinal inflammation has attracted increasing interest in AMD research and the development of new therapeutics. While the precise molecular origins of inflammatory activation in AMD remain elusive, attenuation of the inflammatory pathways via complement inhibition slowed the progression of retinal atrophy (Spaide & Vavvas, 2023). Our data provide support for the concept that retinal immunomodulation, facilitated by the AT‐MSC exosomes can shift the detrimental inflammatory effects induced by Aβ in the retina towards a more favourable outcome. This was evidenced by the preservation of intact ERG responses and improved cell viability. Future studies will elucidate further details of the molecular mechanism and therapeutic potentials of MSC‐exosomes in AMD.

## AUTHOR CONTRIBUTIONS

**Amanda Qarawani**: Formal analysis (equal); investigation (equal); methodology (equal); validation (equal); visualization (equal); writing—original draft (equal); writing—review and editing (equal). **Efrat Naaman**: Formal analysis (equal); investigation (equal); methodology (equal); validation (equal); visualization (equal); writing—original draft (equal); writing—review and editing (equal). **Rony Ben‐Zvi Elimelech**: Investigation (equal). **Michal Harel**: Investigation (equal). **Shahaf Sigal‐Dror**: Investigation (equal); validation (equal). **Tali Ben‐Zur**: Investigation (equal); methodology (equal). **Tamar Ziv**: Formal analysis (equal); investigation (equal); methodology (equal). **Daniel Offen**: Conceptualization (equal); investigation (equal); methodology (equal); project administration (equal). **Shiri Zayit‐Soudry**: Conceptualization (equal); funding acquisition (equal); investigation (equal); methodology (equal); project administration (equal); supervision (equal); visualization (equal); writing—original draft (equal); writing—review and editing (equal).

## CONFLICT OF INTEREST STATEMENT

The authors report no conflicts of interest.

## Supporting information



Supporting Information

Supporting Information
