# Supplementary material for: Mesenchymal stem cell‐derived exosomes mitigate amyloid β‐induced retinal toxicity: Insights from rat model and cellular studies
Source: J Extracell Biol. 2025 Jan 22;4(1):e70024. doi: 10.1002/jex2.70024 (PMC11752158; doi:10.1002/jex2.70024)
Supplement: Supplementary file 1 — Supporting Information [file JEX2-4-e70024-s001.pptx]

## Slide 1
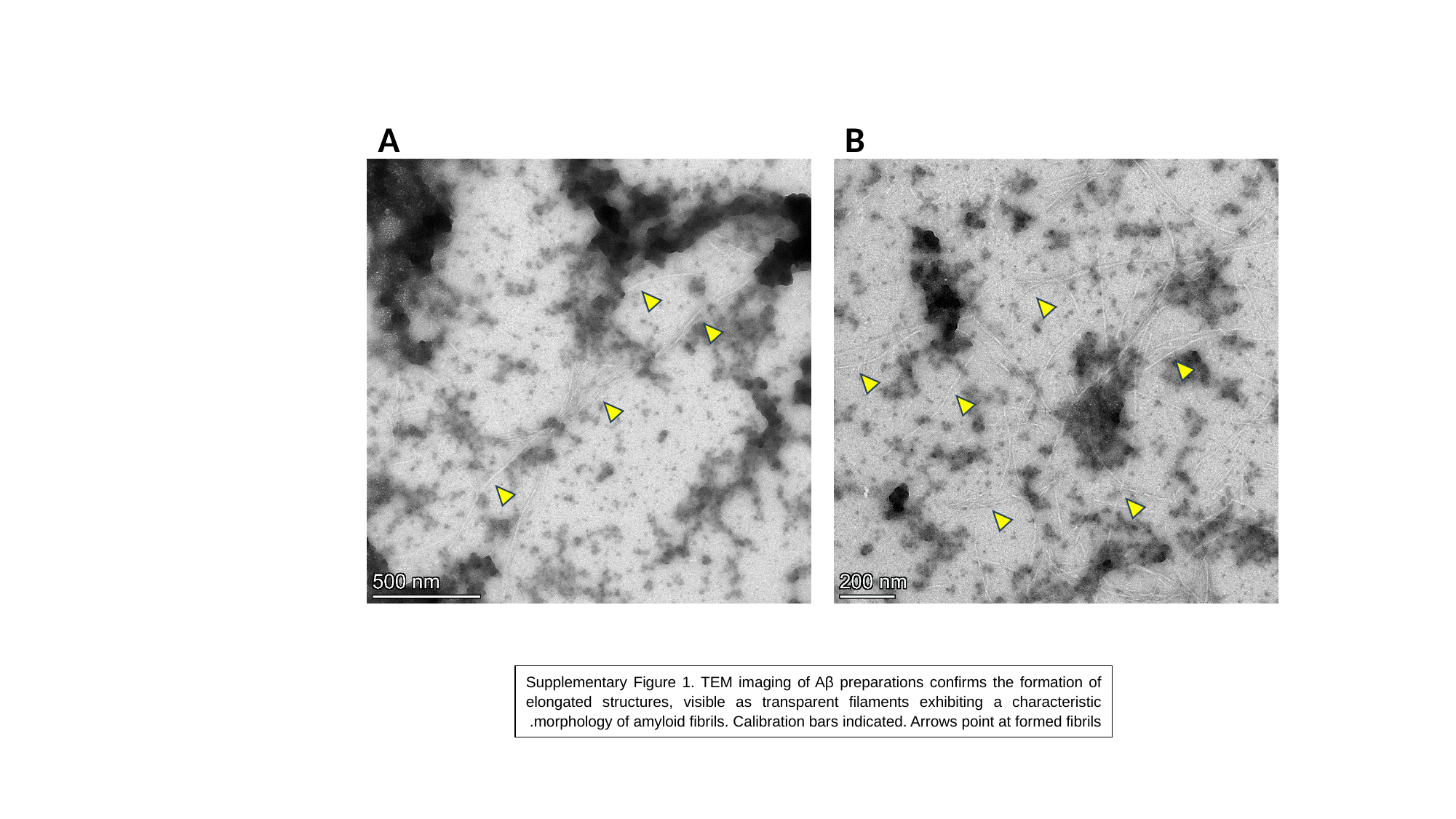

A
B
Supplementary Figure 1. TEM imaging of Aβ preparations confirms the formation of elongated structures, visible as transparent filaments exhibiting a characteristic morphology of amyloid fibrils. Calibration bars indicated. Arrows point at formed fibrils.
