# Supplementary material for: Mesenchymal stem cell‐derived exosomes mitigate amyloid β‐induced retinal toxicity: Insights from rat model and cellular studies
Source: J Extracell Biol. 2025 Jan 22;4(1):e70024. doi: 10.1002/jex2.70024 (PMC11752158; doi:10.1002/jex2.70024)
Supplement: Supplementary file 2 — Supporting Information [file JEX2-4-e70024-s002.docx]

| ***Protein names*** | ***Protein IDs*** | ***Gene names*** | ***Fold Change*** | ***p-value*** |
| --- | --- | --- | --- | --- |
| *BetaA2-crystallin* | Q8CGQ0 | Cryba2 | 0.045 | 0.204 |
| *RAS guanyl-releasing protein 1* | Q9R1K8 | Rasgrp1 | 0.063 | 0.179 |
| *Interferon gamma induced GTPase* | A0A8I5ZSB0 | Igtp | 0.082 | 0.185 |
| *Beta-crystallin A3* | P14881 | Cryba1 | 0.11 | 0.269 |
| *Beta-crystallin B3* | P02524 | Crybb3 | 0.131 | 0.274 |
| *Ubiquitin-conjugating enzyme E2E 2;Ubiquitin-conjugating enzyme E2E 3* | A0A8I6A2G8;F7F5A1 | Ube2e2;Ube2e3 | 0.132 | 0.196 |
| *Beta-crystallin A4* | P56374 | Cryba4 | 0.134 | 0.311 |
| *Alpha-crystallin A chain* | P24623 | Cryaa | 0.15 | 0.264 |
| *Gamma-crystallin B* | P10066 | Crygb | 0.15 | 0.329 |
| *RCG23004, isoform CRA_a* | D3ZN28 | Zfp53 | 0.169 | 0.184 |
| *Gamma-crystallin A* | P10065 | Cryga | 0.17 | 0.306 |
| *Gamma-crystallin C* | P02529 | Crygc | 0.17 | 0.293 |
| *Thymidine kinase 2* | D3ZGQ2 | Tk2 | 0.189 | 0.003 |
| *Beta-crystallin B1* | P02523 | Crybb1 | 0.195 | 0.3 |
| *Collagen type IV alpha 2 chain* | F1M6Q3 | Col4a2 | 0.206 | 0.152 |
| *Coiled-coil domain containing 9* | M0RA86 | Ccdc9 | 0.225 | 0.188 |
| *Premelanosome protein* | D3ZED8 | Pmel | 0.228 | 0.172 |
| *Fibronectin type 3 and SPRY domain-containing protein* | B1H2A2 | Fsd1 | 0.228 | 0.202 |
| *Serine/threonine-protein kinase MARK2* | O08679 | Mark2 | 0.248 | 0.224 |
| *Alpha-crystallin B chain* | P23928 | Cryab | 0.277 | 0.218 |
| *Collagen type IV alpha 1 chain* | F1MA59 | Col4a1 | 0.313 | 0.151 |
| *Cilia- and flagella-associated protein 20* | Q499T7 | Cfap20 | 0.341 | 0.288 |
| *RPTOR independent companion of MTOR, complex 2* | A0A8I5ZNG4 | Rictor | 0.378 | 0.254 |
| *Serine protease inhibitor A3K* | P05545 | Serpina3k | 0.387 | 0.373 |
| *DNA repair protein SWI5 homolog* | Q63ZV7 | Swi5 | 0.406 | 0.001 |
| *Pre-mRNA processing factor 39* | D4A5S9 | Prpf39 | 0.43 | 0.295 |
| *Family with sequence similarity 172, member A* | A0A8I6GH74 | Fam172a | 0.444 | 0.248 |
| *Multivesicular body subunit 12B* | D4A732 | Mvb12b | 0.464 | 0.367 |
| *Collectrin* | Q9ESG3 | Cltrn | 0.531 | 0.116 |
| *N-terminal Xaa-Pro-Lys N-methyltransferase 1* | Q5BJX0 | Ntmt1 | 0.599 | 0.178 |
| *Chloride intracellular channel protein 6* | Q811Q2;Q9EPT8 | Clic6 | 0.629 | 0.094 |
| *Glyceraldehyde 3-phosphate dehydrogenase NAD(P) binding domain-containing protein* | A0A8I6AD02 | ENSRNOG00000069655 | 1.492 | 0.389 |
| *ADF-H domain-containing protein* | D4A315 | AC093995.1 | 1.563 | 0.117 |
| *Transcription factor AP-2 beta;Transcription factor AP-2-alpha* | A0A0G2KAT2;P58197 | Tfap2b | 1.891 | 0.044 |
| *Transforming, acidic coiled-coil containing protein 1* | A0A0G2K9K2 | Tacc1 | 2.632 | 0.019 |
| *Sodium/potassium/calcium exchanger 3 (Fragment)* | Q9EPQ0 | Slc24a3 | 2.739 | 0.306 |
| *ADAMTS-like 2* | D4A4X6 | Adamtsl2 | 2.775 | 0.269 |
| *ArfGAP with dual PH domains 1* | O88768 | Adap1 | 2.778 | 0.257 |
| *Prospero homeobox 1* | D3ZU00 | Prox1 | 3.046 | 0.1 |
| *cGMP-inhibited 3,5-cyclic phosphodiesterase 3A* | Q62865 | Pde3a | 3.057 | 0.150 |
| *Substance-P receptor* | P14600 | Tacr1 | 3.092 | 0.235 |
| *Guanine nucleotide exchange factor MSS4-like;Guanine nucleotide exchange factor MSS4* | A0A8I6GKW6;Q08326 | LOC120100841;Rabif | 3.101 | 0.156 |
| *Cadherin-20* | Q5DWV1 | Cdh20 | 3.128 | 0.179 |
| *Complexin-1* | P63041 | Cplx1 | 3.581 | 0.333 |
| *A-kinase anchor protein 13* | F1M3G7 | Akap13 | 3.796 | 0.067 |
| *Protein spinster homolog 1* | Q2YDU8 | Spns1 | 4.006 | 0.185 |
| *Seizure related 6 homolog like 2* | A0A8I5ZQG6 | Sez6l2 | 4.55 | 0.181 |
| *Cytochrome c oxidase subunit 5A, mitochondrial* | A0A8I6A0L0 | LOC100361008 | 4.585 | 0.244 |
| *CCR4-NOT transcription complex subunit 9* | Q5PQL2 | Cnot9 | 4.699 | 0.247 |
| *Zinc finger protein 830* | Q3MHS2 | Znf830 | 5.002 | 0.184 |
| *Butyrophilin, subfamily 3, member A2* | F7F1D5 | Btn3a2 | 5.378 | 0.158 |
| *Dynein axonemal heavy chain 5 (Fragment)* | M0R8U1 | Dnah5 | 6.626 | 0.189 |

Supplementary Table 1. The differentially expressed proteins in the rat's retina. Rats (n=3) treated with fibrillar Aβ injections with prior MSC-exosome administration vs fibrillar Aβ alone and sacrificed 5 days after Aβ injection. All identified proteins had at least 2-fold change difference in at least 2 biological repeats. Statistical analysis was performed using paired student’s t-test. ** indicate p value < 0.05.
